# When Kinesthesia Becomes Visual: A Theoretical Justification for Executing Motor Tasks in Visual Space

**DOI:** 10.1371/journal.pone.0068438

**Published:** 2013-07-05

**Authors:** Michele Tagliabue, Joseph McIntyre

**Affiliations:** Centre d'Etude de la Sensorimotricité, (CNRS UMR 8194), Université Paris Descartes, Institut des Neurosciences et de la Cognition, Sorbonne Paris Cité, Paris, France; VU University Amsterdam, Netherlands

## Abstract

Several experimental studies in the literature have shown that even when performing purely kinesthetic tasks, such as reaching for a kinesthetically felt target with a hidden hand, the brain reconstructs a visual representation of the movement. In our previous studies, however, we did not observe any role of a visual representation of the movement in a purely kinesthetic task. This apparent contradiction could be related to a fundamental difference between the studied tasks. In our study subjects used the same hand to both feel the target and to perform the movement, whereas in most other studies, pointing to a kinesthetic target consisted of pointing with one hand to the finger of the other, or to some other body part. We hypothesize, therefore, that it is the necessity of performing inter-limb transformations that induces a visual representation of purely kinesthetic tasks. To test this hypothesis we asked subjects to perform the same purely kinesthetic task in two conditions: INTRA and INTER. In the former they used the right hand to both perceive the target and to reproduce its orientation. In the latter, subjects perceived the target with the left hand and responded with the right. To quantify the use of a visual representation of the movement we measured deviations induced by an imperceptible conflict that was generated between visual and kinesthetic reference frames. Our hypothesis was confirmed by the observed deviations of responses due to the conflict in the INTER, but not in the INTRA, condition. To reconcile these observations with recent theories of sensori-motor integration based on maximum likelihood estimation, we propose here a new model formulation that explicitly considers the effects of covariance between sensory signals that are directly available and internal representations that are ‘reconstructed’ from those inputs through sensori-motor transformations.

## Introduction

A number of previous studies have suggested that the CNS plans and executes targeted movements of the hand using a visual representation of the movement even when the target is presented kinesthetically (e.g. pointing with one hand to the other) and even when no visual feedback about the hand is allowed [1]–[5]. This is in apparent contrast with our own studies on human sensori-motor integration [6], [7] in which we observed that if subjects were asked to align their hidden hand to the orientation of a kinesthetically felt target, they completely ignored the information related to the visual scene, indicating that the brain executes purely kinesthetic tasks (K-K: kinesthetic target and kinesthetic response) without using a visual representation of the movement. This apparent contradiction, however, could be related to a fundamental difference between the motor tasks that the subjects were asked to perform in these different sets of studies. Whilst in our study subjects felt and reproduced the target position/orientation with the same hand, participants in the other aforementioned studies had to sense the target with the left hand or with a foot, hidden under a table, and reproduce its position with the right hand. Based on this observation, we postulated that the use of a visual representation of the movement could be related to the necessity of performing an inter-limb transformation of the kinesthetic information.

**Figure 1:**
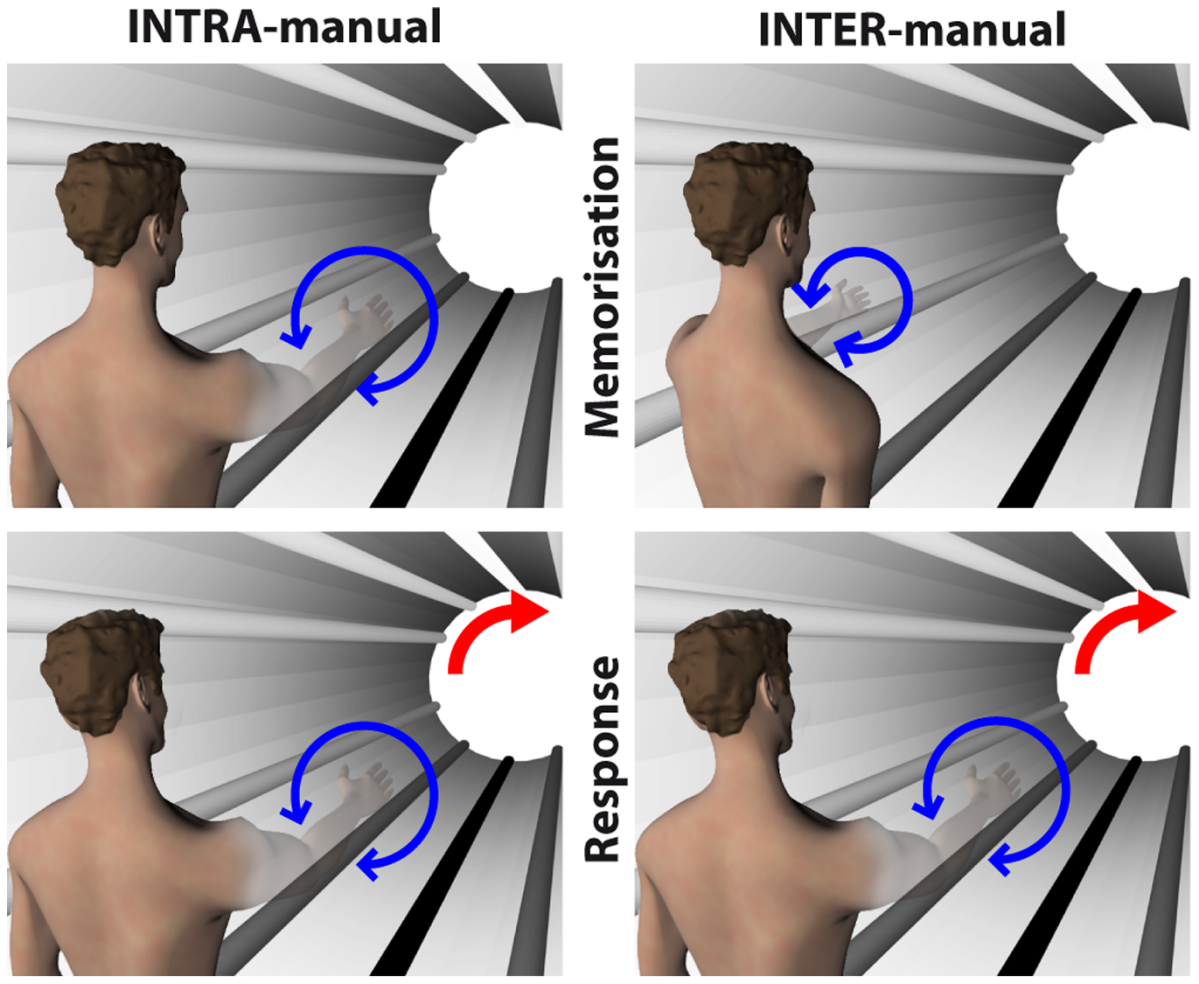
Experimental conditions. In both INTRA- and INTER-manual condition subjects felt the orientation of a kinesthetic target and reproduced it by prono-supinating the hand (blue arrows). Subjects never saw their hand, thanks to a virtual reality system. Whilst in the INTRA condition the subject sensed the target and reproduced the orientation with the same (right) hand, in the INTER condition targets were sensed with the left and reproduced with the right. To detect the use of visual representation of the task, in some of the trials the virtual scene imperceptibly rotated with respect to gravity (red arrows).

We set out to test this hypothesis by asking healthy volunteers to reproduce, by pronating/supinating their unseen hand, a kinesthetically memorized orientation (i.e. in a replication of the K-K condition of our previous study [6]) and we compared their responses in two different conditions (Figure 1). In the first, called INTRA-manual, subjects memorized and reproduced the orientation with the same hand. In the second, called INTER-manual, subjects memorized the orientation with one hand and responded with the other. Note that in the INTER-manual task, subjects could have simultaneously sensed the target with one hand and reproduced the target orientation with the other. We chose instead to have subjects reproduce the target from memory even in the bilateral task, so that any differences observed between INTER and INTRA could not be attributed to differing memory requirements of the tasks.

In both INTER and INTRA, we exploited the peculiarity of our reach-to-grab paradigm in virtual reality that allows us to assess the weight given to visual information even when the subject had only kinesthetic sensory inputs to control the movement [6], [7]. Specifically, subjects were asked to tilt the head laterally after the target memorization and during the head movement of half of the trials we introduced an imperceptible sensory conflict between the orientation of the visual scene and the direction of gravity. Under these circumstances, deviations of the final hand orientation should be proportional to the relative weight given to the visual-scene versus gravito-kinesthetic information used to control the movement. If our hypothesis is correct, we expect visual encoding to play a role – and hence we expect to see a deviation of the responses due to the visual scene tilt – in the INTER-manual, but not in the INTRA-manual task.

In an effort to explain our results, we applied models of optimal sensory-motor integration based on the maximum likelihood principle (MLP), which falls in the domain of Bayesian optimal estimation [8]–[16]. Recent models assume that to control goal-directed upper-limb movements the brain evaluates the difference between target and hand concurrently in visual and kinesthetic reference frames [4], [6], [7], [17]. When direct sensory information is not available in one or more sensory modalities, internal representations of the target or hand may be ‘reconstructed’ via coordinate transformations, through recurrent neural networks in the parietal cortex (for review see [18]). Thus, when reaching a visual target with an unseen hand, the CNS might, for instance, reconstruct a visual representation of the hand to be compared with the available sensory information about the target. The relative weight given to the comparisons performed in visual or kinesthetic space would be determined by the expected variability within each representation, taking into account the variability added by any sensori-motor transformations that may be required [14], [19]–[21].

**Figure 2:**
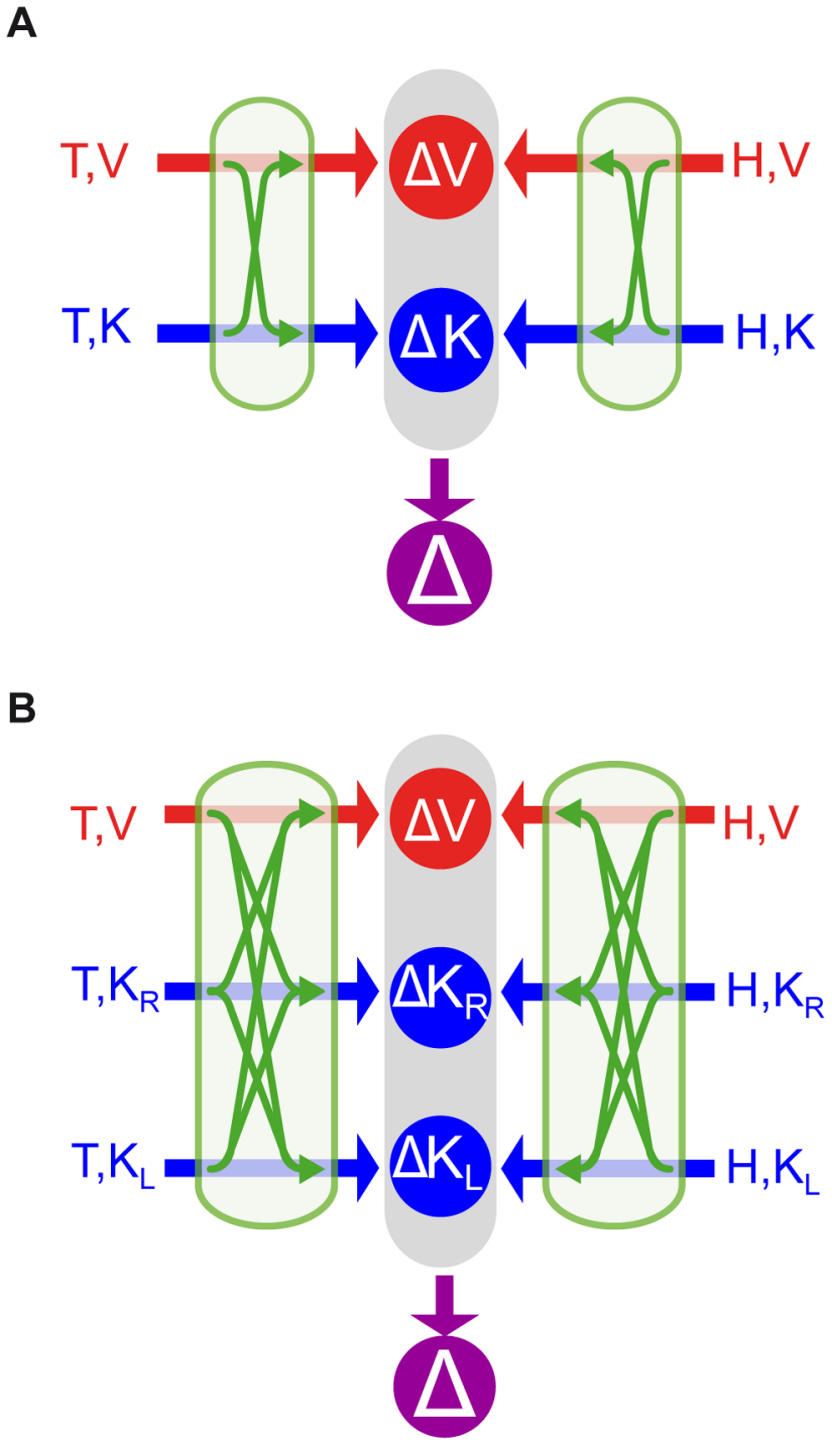
Models of sensory-motor integration. Both models A and B assume that the brain can perform concurrent visual and kinesthetic comparisons (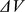, 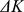) between the target (T) and the hand (H) and optimally combine the results of these comparisons to estimate the target-hand distance (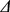). Both models also include the possibility of performing sensory reconstructions of the information about the target and the effector, as represented by the green arrows. Model A does not distinguish between the kinesthetic information from left and right limb and they can be compared directly, therefore two concurrent comparisons are possible (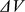, 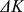). For model B kinesthetic information about the target and the response can be directly compared only if they come from the same limb. Therefore this model explicitly differentiates three concurrent references frames for the comparisons: visual (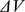), kinesthetic linked to the right limb (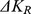) and kinesthetic linked to the left limb (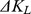).

In the context of this model, we asked whether kinesthetic information from the two hands is encoded in a single, kinesthetic, perhaps trunk-centered, reference frame, allowing them to be directly compared, or whether the kinesthetic information from the right and left arms is encoded in two different, arm-specific reference frames, perhaps centered on each shoulder [22], and thus requiring sensory transformations between them. For each of these two alternatives we proposed the corresponding mathematical formulation of the model (Figure 2), and we tested their ability to predict subjects' performance on each task. We then considered the effects of co-variance between transformed sensory signals as a means of reconciling the predictions of MLP with our experimental observations and with our hypothesis that the brain reconstructs concurrent movement representations only if a direct target-hand comparison is not possible [6].

## Results

**Figure 3:**
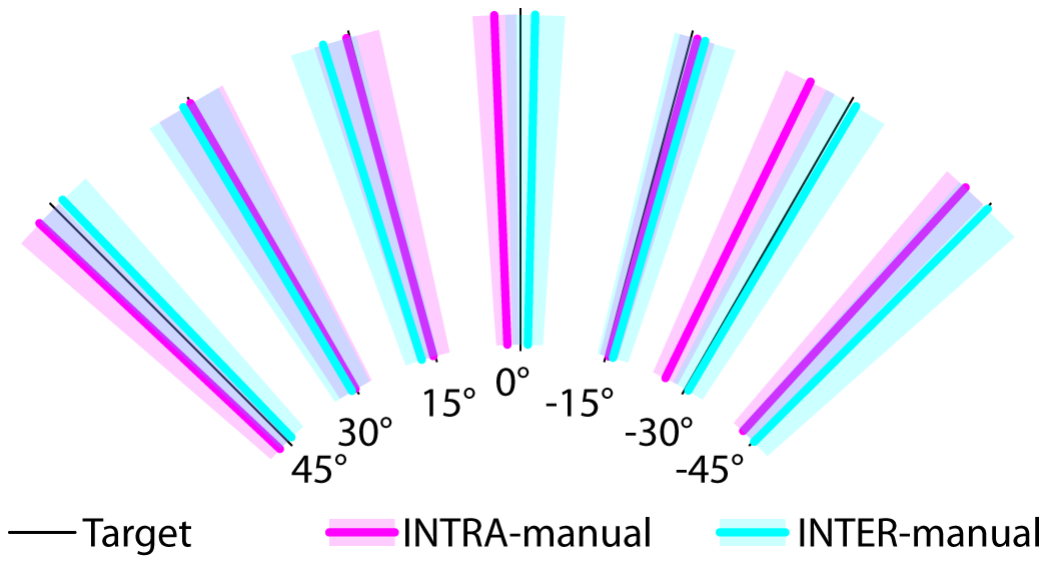
Subject responses to each target orientation in the INTRA- and INTER-manual conditions for the trials without conflict. Thick lines are the average responses (transparent areas’ width represent the standard error) of all subjects, combining trials with left and right head tilt as explained in the methods.

**Figure 4:**
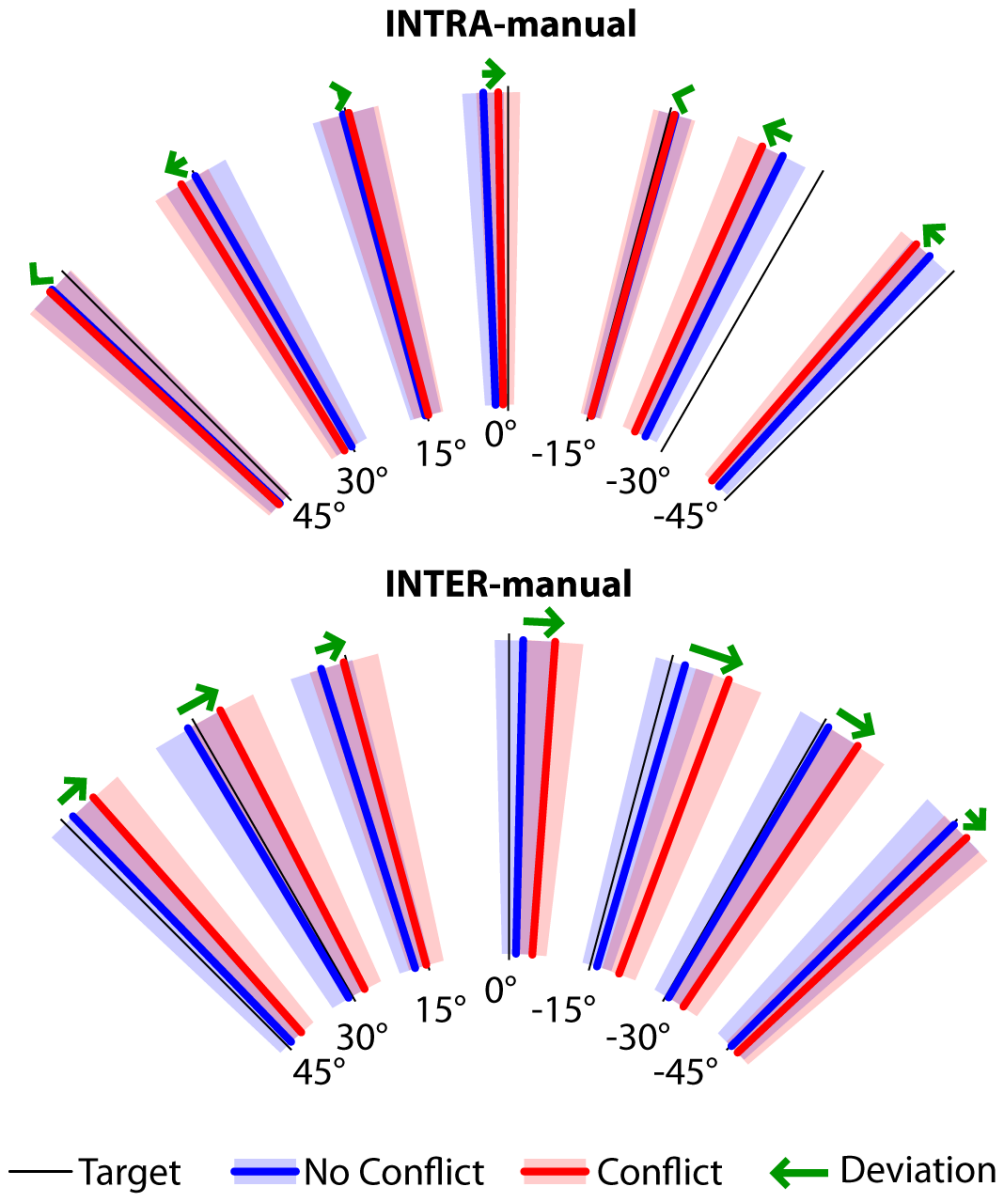
Subject responses to each target orientation in the INTRA- and INTER-manual conditions. The responses for the trials with and without conflict are represented separately. Thick lines are the average responses (transparent areas’ width represent the standard error) of all subjects, combining trials with left and right head tilt as explained in the methods. Green arrows represent the measured responses’ deviations due to the tilt of the visual scene.

**Figure 5:**
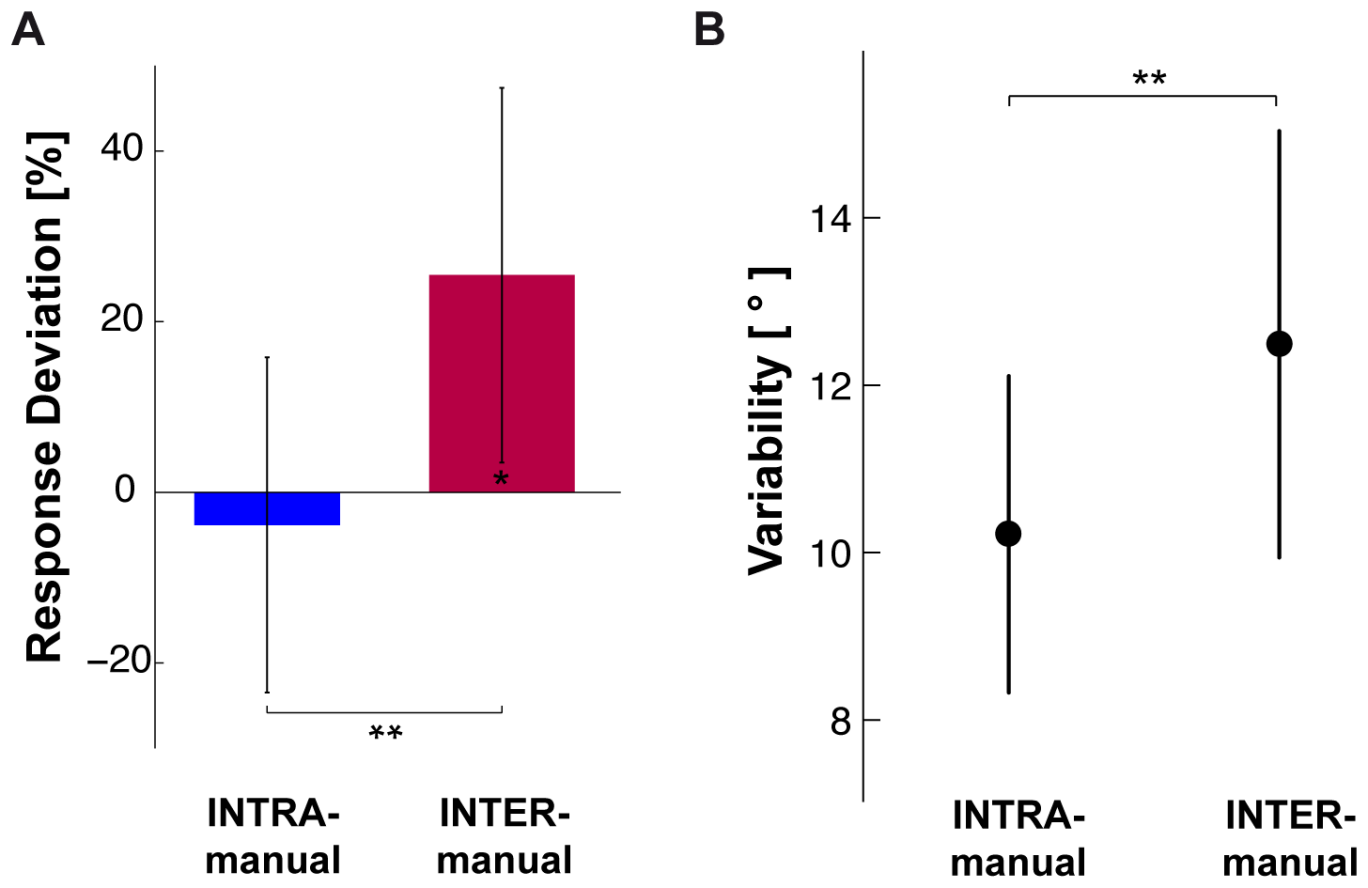
A. Experimental results for the deviation of responses induced by the imperceptible tilt of the visual scene and the variability of responses in the INTRA- and INTER-manual conditions. Results are expressed as a percentage of the theoretical deviation expected if subjects aligned the response with respect to the visual scene. B. Average within-subject variability (standard deviation) of the response without conflict. In both panels vertical whiskers represent the 0.95 confidence intervals. Stars represent the significance of the main effects in the ANOVA and the results of the t-test comparison with the nominal 0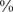 value. (** 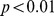; * 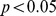).

Figure 3 shows for the INTRA and INTER-manual conditions the average responses to each target orientation in trials without conflict, which do not differ appreciably between the two experimental conditions: statistical analyses on the aligning errors showed no significant effect of the experimental condition (ANOVA F(1,15) = 2.13, p = 0.17) or interactions between condition and target orientation (ANOVA F(6,90) = 0.92, p = 0.49). On the other hand, clear differences can be observed in Figure 4 for the responses in the trials with conflict: in the INTRA condition the responses were not consistently deviated by the conflict, in the INTER condition conflict caused the responses to all target orientations to be deviated in the same direction. These latter observations were confirmed by the statistical analysis of the global deviation of the responses. Figure 5A shows that in the INTER-manual condition responses were significantly deviated by the inclination of the visual surround (one-tailed t-test with respect to 0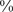: t(15) = 2.27, p = 0.02), whilst no statistical difference from the null deviation could be detected in the INTRA-manual condition (one-tailed t-test with respect to 0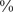: t(15) = −0.37, p
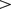
0.25). The differing effects between the two experimental conditions was confirmed by a significant difference between the INTRA- and INTER-manual conditions (ANOVA F(1,15) = 8.81, p = 0.009). The comparison of the response variability between the two experimental conditions, reported in Figure 5B, shows that subjects were more precise when they had to reproduce an orientation felt with the same hand than with the other hand (ANOVA F(1,15) = 10.38, p = 0.005).

### Theoretical Modeling

Given the clear empirical observations shown above, we then undertook a mathematical analysis to better understand the implications of these results for recent theories of sensorimotor integration. To represent the performance of this task, we considered the two candidate models shown in Fig. 2, both of which assume that the brain performs concurrent target-hand comparisons in different sensory modalities and then optimally combines the result of these comparisons to create the movement vector (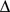). For model A, 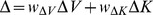. For model B 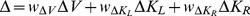 (for details about the notations, see the caption to Figure 2). Both models also assume that the brain can reconstruct missing information from available signals through recurrent neural networks (for review see [18]). To make predictions using these two models, we hypothesized that the weighting (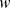) of each individual comparison would be determined by the maximum likelihood principle, which states that the two or three quantities computed in each case will be combined according to the relative variance of each signal (see Methods). We assumed that sensorimotor transformations add variability [19]–[21], such that reconstructed signals will have greater variance than the source signal. Note that this is a slight simplification. A transformation might literally add stochastic noise if the transformation involves the integration of the primary signal with other, noisy sensory inputs. But transformations might also amplify the variability of the primary signal if they included distortions, perceptual biases or other non-linearities. We assume here, however, that on a target-by-target basis the effects of such distortions will be small, such that the effects of a sensorimotor transformation on the variability of the transformed output can be adequately represented by the simple addition of variance and that the MLP equations can be applied directly to the sum, as was done in other sensori-motor integration models [4], [14]. We hence computed relative weights between directly sensed and reconstructed comparisons based on the assumption that transformations add variance [6].

**Figure 6:**
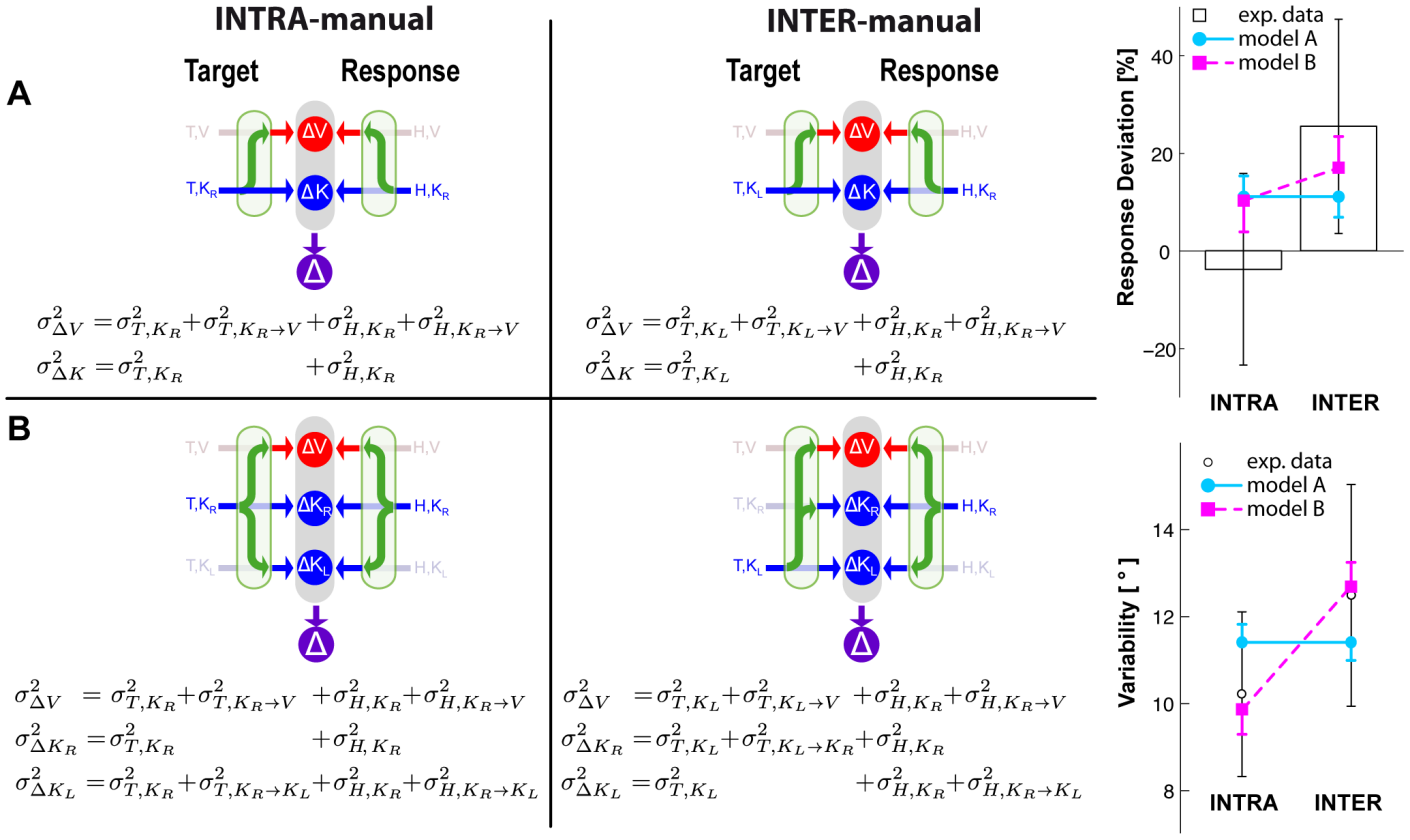
Model predictions when MLP is applied while ignoring the covariance between direct and reconstructed sensory signals. On the right, graphical representations of the information flow for model A and B and for the INTRA and INTER conditions, together with the equations of the variance corresponding to each concurrent target-hand comparison. The best-fit Model A predicts equal weight given to visual versus kinesthetic information between the INTRA and INTER tasks, and the same response variability in both cases. Model B predicts a greater weighting of visual information and higher response variability for the INTER task, qualitatively similar to the empirical data, but does not correctly predict a zero weighting of visual information in the INTRA-manual task.

With few exceptions [23], all studies in Neuroscience to date have, to our knowledge, applied the maximum likelihood equations as if the original and reconstructed signals are independent (i.e. uncorrelated). To demonstrate the limitations of that approach, and to gain insight into how covariance might affect the results, we first computed the model predictions without taking into account the covariance between the original sensory inputs and internal representations that are computed through sensorimotor transformations of those signals. Applying Eqs. 4 and 5 from the Methods for model A we have:
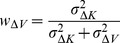
(A1)

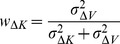
(A2)where 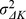 and 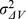 are the variance associated to the kinesthetic and visual comparison respectively. For model B, applying Eqs 6–8 from the Methods gives:
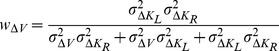
(B1)

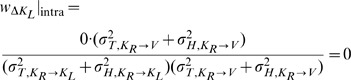
(B2)

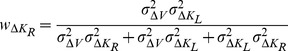
(B3)where 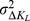, 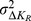, and 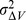
are the variances associated to the left-hand kinesthetic, right-hand kinesthetic and visual comparison respectively. The net variance of each of the comparisons, 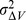, 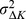, 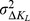 and 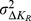, depends on the variance of the input signals (e.g. 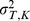, the variance of target orientation as measured via kinesthesia) and the variance added by any sensorimotor transformations required to reconstruct an internal representation (e.g. 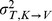, representing the variance added when transforming target information from the kinesthetic to visual domains, including the variability of the sensory information that allows one to measure the orientation of the head, such as the visual scene, vestibular signals and neck proprioception). For the detailed equations defining the variance of each comparison, depending on the experimental conditions and hypotheses, see Figure 6. In order to reduce the number of parameters of the model and allow a meaningful fitting to the experimental data, the following assumptions were made:

- The variability of the kinesthetic information from the left and right arm was similar [24]: 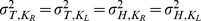.
- The variance associated to the inter-limb transformation of kinesthetic information about the target and about the response was the same (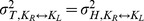).
- The variance associated to the reconstruction of visual information from kinesthesia from either the right or left arm was the same (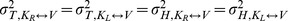).

Note that asymmetries in errors when matching kinesthetic or visual targets with the right or left hand [25] mean that the last of these three assumptions may not be entirely true. We nevertheless adopted these assumptions so as to reduce the number of free parameters to three (variance of the kinesthetic signals 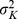, variance due to inter-limbs transformations 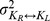 and variance due to visuo-kinesthetic transformations 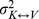) whose values could be determined to best fit the experimentally observed values of response deviation and response variability that are reported in Figure 5.

Figure 6 shows the model predictions based on equations A1–A2 and B1–B3, i.e. when co-variation between sensory signals and reconstructed internal representations is ignored. To the left we show a 2×2 grid, corresponding to the predictions of each of the two models (A and B) for each of the two experimental conditions (INTRA and INTER). Within each model we show the sources of variance associated with each individual comparison necessary for equations A1–A2 and B1–B3, including the noise associated with input signals that are present and variance added by any sensory transformations that may be required. Sensory inputs that are not available directly are grayed out in the schematic diagrams. To the right we superimpose on the experimental data the predictions of each of the two models with weights calculated from equations A1–A2 and B1–B3 that best fit all of the data (deviations and variability). The estimated variability associated with the kinesthetic information and each transformation as a result of the best-fit procedure are shown in Table 1.

**Table 1:** Values (expressed in °) of the predicted variability (±95% confidence interval) associated with the kinesthetic information (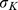), inter-limb kinesthetic transformations (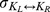) and visuo-kinesthetic reconstructions (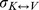) that best fit the experimental data for the models in Figure 2.

	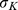	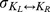	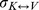
Model A	8.5 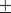 0.3	–	22.8 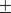 14.5
Model A′	8.1 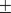 0.3	–	–
Model B	8.1 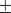 0.5	16.0 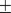 3.5	20.2 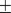 14.7
Model B′	7.2 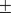 0.3	17.2 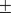 1.6	17.2 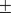 1.8

From this analysis one can eliminate Model A as an explanation of the empirical data. According to this model, there is no difference between comparing the posture of the right hand to the left hand versus comparing the right hand’s posture with itself. Thus, this model predicts precisely the same weight given to the visual comparison, and thus precisely the same deviations of the response due to tilt of the visual scene between the INTER and INTRA tasks. Model A also predicts that the variability of responses will be equal between the INTER and INTRA tasks. We observed, however, a statistically-significant difference in both deviation and variability between the INTER and INTRA conditions, inconsistent with these predictions (two-tailed t-test between Model A’s prediction and the experimentally observed difference for response deviations between the INTER and INTRA conditions: t(15) = 3.0, p
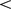
0.01).

Model B does a better job of capturing the qualitative features of the measured data. According to this model, comparing the posture of the left hand to the right hand requires inter-limb sensory transformations. Thus, the kinesthetic comparisons will be more variable in the INTER task compared to the INTRA task. This allows the model to predict a different weighting between visual and kinesthetic information for the INTER vs. INTRA tasks, meaning that the deviations induced by rotations of the visual scene and the overall variability of responses are expected to be greater in the INTER task than in the INTRA task. Nevertheless, Model B, with weights computed to best fit the data, predicts a smaller differential of the response deviations between the INTRA and INTER tasks than what we actually observed (two-tailed t-test between Model B's prediction and the experimentally observed difference in response deviations between the INTER and INTRA conditions: t(15) = 2.2, p
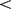
0.05). Indeed, using reasonable assumptions about the relative amounts of variability in each sensory signal and in each sensory reconstruction, one cannot expect to see zero weight given to a visual comparison in the INTRA condition using Model B, even though that is what we observed experimentally.

This inability to reproduce the deviation data with Model B was insensitive to the precise values of response variability used to perform the fitting. The only way that one could expect to see the observed difference in deviations between INTER and INTRA with Model B would be if the difference in response variability between INTRA and INTER was much greater than what was observed. But to achieve the better than 7∶1 ratio that would be required to reproduce the deviation data with Model B, either the overall response variability would have to be much smaller (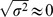) in the INTRA condition or much larger (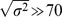) in the INTER condition than the actually observed values (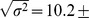 1.9 for INTRA and 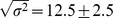 for INTER).

**Figure 7:**
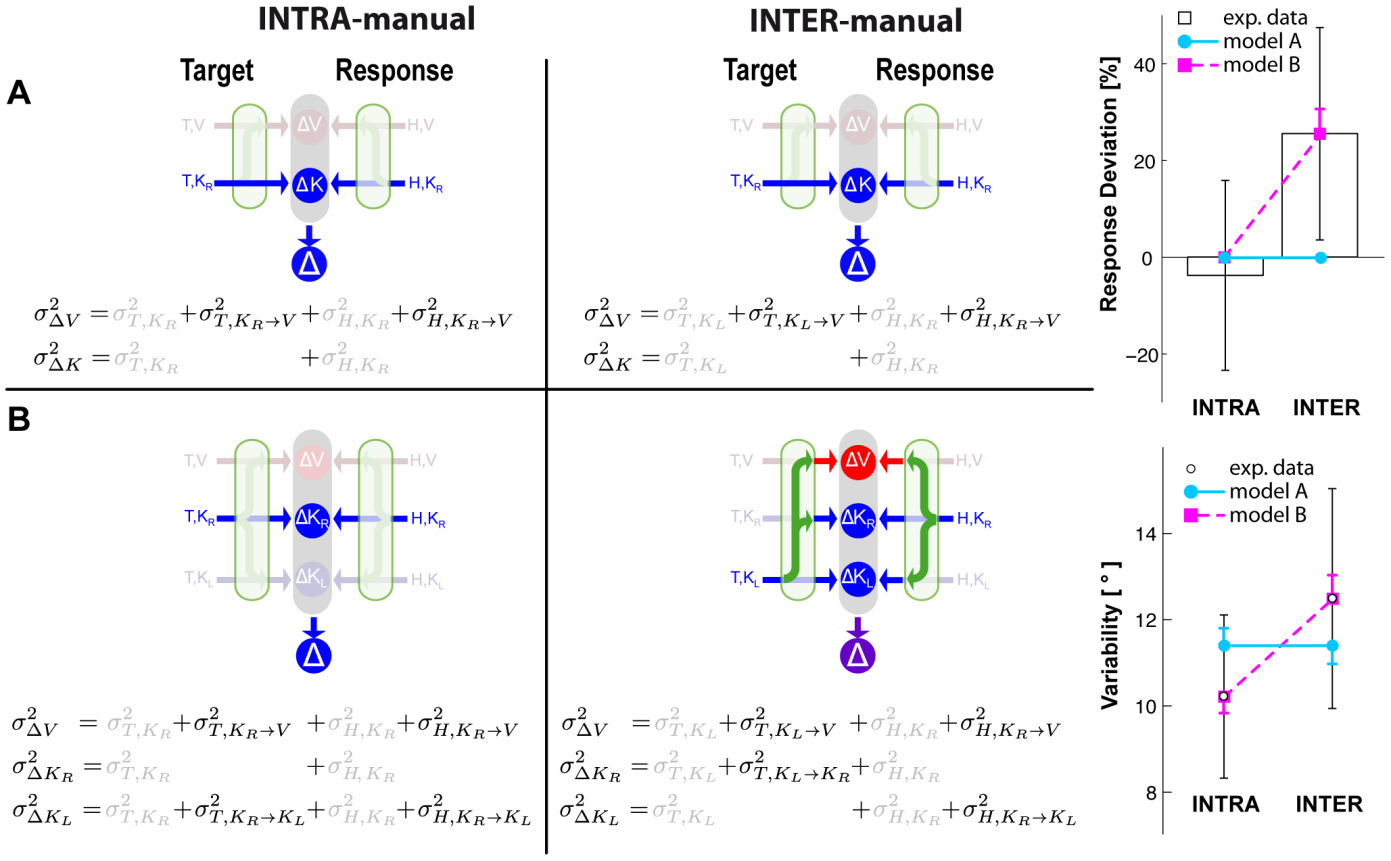
Model predictions when covariance is taken into account in the calculation of the optimal weights for each individual comparison. On the right, graphical representations of the information flow for Model A’ and B’ and for the INTRA and INTER conditions, together with the equations of the variance corresponding to each concurrent target-hand comparison. Components of variance common to all branches, and hence not used to define the relative weights for 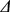, are grayed out. Model A’ predicts that there will be no reconstruction of the task in visual space for either INTRA or INTRA, because there is no variance in the 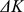 comparison that is not included in the 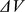 comparison. Deviation of the response with visual scene tilt should be 0 and the variance of the response should be the same for both conditions. Model B’ predicts that the task will be carried out only in kinesthetic space for the INTRA condition, but that both kinesthetic and visual comparisons will be made in the INTER condition. Only Model B’ can accurately fit the data.

To adequately fit both the deviation and variability data we needed to take into account the covariance between a reconstructed signal and its source when computing the MLP weights. As explained in the Methods section, this means that the relative weighting of the parallel comparisons is based on the variance of the independent components of each comparison, neglecting the variance of the common components. In Figure 7 we therefore report the variance associated to each possible comparison with the component of the variance common to all branches grayed out.

For model A we note that there is no component of variance in kinesthetic comparison 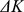 that is not also included in the visual comparison 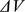. Applying Eqs. 16–17 of the Methods, the predicted weight is zero for the visual comparison and one for the kinesthetic comparison for both INTER and INTRA conditions:
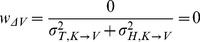
(A'1)

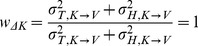
(A'2)


Because both conditions rely on 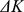 only, one should also observe equal variance of responses between the two conditions. The statistically significant differences of deviations and variability between INTER and INTRA, and the statistically non-zero weight given in to visual information in the INTER condition mean that Model A should be rejected even if covariance is taken into account (two-tailed t-test between the predictions of A’1–A’2 and the experimentally observed difference for response deviations between the INTER and INTRA conditions: t(15) = 3.0, p
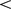
0.01).

On the other hand applying the same concepts for Model B does predict different results between the INTER and INTRA conditions. As shown in Figure 7, for the INTRA situation, there is no variance associated with the direct intra-manual comparison 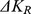 that is not also included in 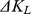 and 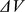. Applying Eqs. 18–20 to this situation gives the following weights for the three concurrent comparisons:
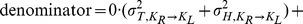


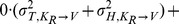


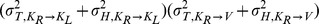


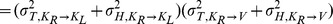


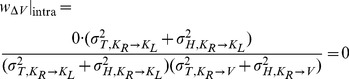
(B'1)

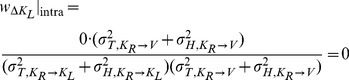
(B'2)

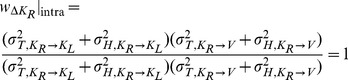
(B'3)


No weight will be given to either 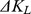 or 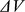 and thus, the deviation of the responses in the INTRA condition is predicted by Model B to be strictly zero. On the contrary, Figure 6 shows that in the INTER condition each of the comparisons 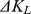, 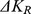 and 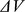 contains a component of variance that is not included in the two others, because each comparison requires at least one transformation, the transformations are all different, and each of these transformation adds variability that is independent from the others. In this case applying Eqs. 18–20 predicts that some non-zero weight will be given to each of the three comparisons to determine the optimal outcome.
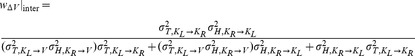
(B'4)

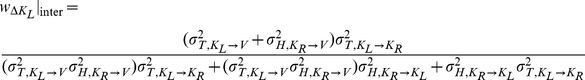
(B'5)

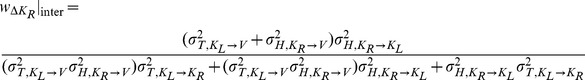
(B'6)


The estimated variances associated with the kinesthetic information and sensory transformations as a result of the best-fit procedure are shown in Table 1. The non-zero weight given to 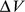 means that Model B does predict a deviation of responses due to tilt of the visual scene. Unlike for the predictions of Eqs. B1–B3, predictions made by taking into account the covariance when applying MLP (Eqs. B’1–B’6) are not statistically different from the empirically estimated difference in weights between the INTER and INTRA conditions (two-tailed t-test between the predictions of Model B’ and the experimentally observed difference for response deviations between the INTER and INTRA conditions: t(15) = 0.4, p
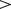
0.70). The variability added by the different transformations also means that the final variance of responses is expected to be greater for the INTER condition, compared to the INTRA condition, which is what we observed. Equations B’1–B’6 are therefore able to fit the salient features of the empirical data (deviation of responses in INTER but not INTRA, different variability between INTER and INTRA).

## Discussion

The results show that, for a purely kinesthetic task of reproducing a kinesthetically sensed orientation with an unseen hand (K-K condition), the brain gives significant weight to visual information when the task requires an inter-limb information transmission (INTER condition), but not when subjects memorized and responded with the same hand (INTRA condition). The lack of a significant effect of visual information in the INTRA condition matches our previous results [6] and is coherent with studies of reaching movements in which subjects also used the same arm to feel the target and to reproduce its remembered position [26]–[29]. The use of visual encoding in otherwise purely kinesthetic pointing tasks has nevertheless been observed in a number of studies that have required comparing one limb to another [1], [4], [5]. Our results suggest that the use of visual encoding in these studies was most likely due to the bilateral nature of the task and that responses would have been different if the subjects had used the same limb to feel the target and to reproduce its position.

In our previous work [6] we concluded that the CNS avoids reconstructing sensory information whenever a direct target-effector comparison is feasible and that if a sensorimotor transformation cannot be avoided, the CNS creates concurrent representations of the task in multiple reference frames potentially tied to other sensory modalities. Based on the reconstruction of a visual representation in the INTER condition observed here, we therefore postulate that inter-manual comparisons require sensorimotor transformations and are therefore not ‘direct’. Independent support for this hypothesis can be found in the literature. Baud-Bovy and colleagues [27] showed that variable errors are oriented toward the left or the right shoulder depending on the arm used to memorize the kinesthetically-presented target, suggesting the existence of distinct reference frames tied to each arm. A transformation would therefore be required to compare the position of the two hands. Evidence for arm-specific reference frames for the control of pointing movements to visual targets has also been reported [19], [22], [30], [31]. The recreation of the task in multiple, alternative reference frames has been reported for other tasks as well. Just as we saw a reconstruction of the task in an alternative (visual) reference frame in the INTER, but not in the INTRA condition, Rao and colleagues [29] reasoned that tactile information plays more of a role in a bilateral reaching task, as compared to a unilateral one, because the bilateral tasks requires a transformation anyway, and so it made sense to transform the task into tactile space as well. Subjects did use an eye-centered reference frame to encode the kinesthetic information even in a unilateral kinesthetic matching task [32], but those subjects verbally reported whether one passive movement imposed by a robot was more to the left or to the right of another. Given that ‘left’ and ‘right’ refer to directions that are not intrinsically defined by the kinesthetic receptors of the arm, sensory transformations would nevertheless be required to verbalize the response. Thus, the need for at least one transformation, or not, appears to be the key factor in determining if additional representations of the task (visual or otherwise) are constructed. This concept explains, in a parsimonious way, the difference between INTER and INTRA reported here and a wide variety of findings reported in the literature.

Avoidance of sensory transformations, including inter-manual transformations, can also explain how the CNS chooses one sensory input over another when sensory information is available simultaneously in more than one modality. In an orientation-matching paradigm [6], [7], where we compared movements to a visual target with only visual feedback (V-V) or with both visual and kinesthetic feedback (V-VK) of the hand, we observed very similar results between these conditions both in terms of global variability and strength of the oblique effect (for review about oblique effect see [33]), suggesting a similar importance given to visual information in both conditions. Helms Tillery also observed similar variable error between a VK-V and V-V condition in a 3D pointing task [20]. Similarly, compared to a unilateral kinesthetic-only task (K-K), we saw no evidence for a reconstruction in visual space when visual information about the hand was added (K-VK). But other studies *have* reported the use of visual information in K-VK tasks [2]–[4], [8], [9], [34]. In those studies, however, subjects did not use the same arm to sense the target and produce the movement. Transforming the kinesthetic target to be compared with visual feedback about the hand would, in the bilateral case, appear to be advantageous. Conversely, adding kinesthetic information about a visual target had little effect in a study by Sabes that compared V-VK and bilateral VK-VK conditions [17]. Generally speaking, eye-centered representation of the movement dominate over kinesthetic cues when subjects reach for visual targets [35], [36]. Indeed, neural activation in the posterior parietal cortex of monkeys during reaching toward visual targets with the hand in view suggests that a target-hand comparison is performed in retinal space, without integration of kinesthetic information about the limb [37].

MLP can explain why the CNS gives greater weight to direct versus reconstructed comparisons if one takes into account the additional variability inherent to transformed signals [14], [19]–[21]. As a direct evidence supporting this idea, Burns and collegues [14] showed that for reaching to a visual target (V-VK) increasing the variability of cross-modal transformations by tilting the head [6], [38] increased the weight given to the direct visual comparison. It is worth noticing, therefore, that subjects in our experiments showed greater variability for INTER than for INTRA (Fig. 5B). A similar increase in variability was observed when contrasting cross-modal conditions (V-K or K-V) to conditions allowing a direct visual or unilateral kinesthetic comparison (V-V and K-K, respectively) [6]. The greater variability in the INTER condition observed here and elsewhere [39], can therefore most likely be ascribed to the variability added by inter-limb transformations, be it through added noise from sensory signals required for the transformation, or due to distortions or non-linearities in the transformation itself. In this vein, the loss of precision when tactile information was compared between limbs [40], [41] has been been attributed to the inter-hemispheric relay [42]–[44] or to the coordinate transformations that would be required to compare stimuli to different fingers within the same hand [45]. In sum, sensorimotor transformations in general, and inter-limb transformations in particular, appear to add variability. According to MLP, performing additional comparisons based on transformed information would do little to improve performance when a direct comparison is possible.

Simply considering the variability added by inter-limb comparisons was not sufficient, however, to properly predict the experimental results within the context of our models. We could only predict the total lack of effect of scene tilt in the INTRA condition by specifically considering the correlation between reconstructed and direct sensory information. More precisely, we showed that if a direct target-response comparison was possible (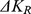 in the INTRA-manual condition), combining it with other comparisons (e.g. 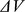) reconstructed from the available kinesthetic information could not reduce response variability. On the other hand, in our INTER condition, where a direct target-effector comparison was not possible, all possible comparisons required some sort of sensory transformation. If the variability of each transformation is independent from the others, combining them can result in a decrease of the overall response variability. Accordingly, subjects reconstructed a visual representation of the task (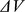), even though just one of the kinesthetic comparisons (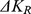 or 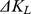),would have been sufficient. These results are in line with our working premise that once a transformation becomes inevitable, a broader slate of redundant comparisons are automatically performed [6]. Furthermore, explicitly taking into account the covariance of transformed signals in the application of MLP provides a firm theoretical basis that explains not only when, but also why the CNS would reconstruct a visual representation of a kinesthetic task [1]–[5].

In conclusion, the fact that in the intra-manual task no role of visual information could be detected in our experiments demonstrates that the brain prefers direct comparisons whenever possible. We have shown that this is because additional reconstructed representations would strongly correlate to the direct comparison and hence would not reduce movement variability. On the other hand, when a sensory transformation is necessary to compare the hand and target position, even if it is just the transformations required to compare one arm to the other, the brain reconstructs the movement in multiple reference frames, thus creating a visual representation of a purely kinesthetic task.

## Methods

### Ethics Statement

The experimental protocol was approved by the IRB of University Paris Descartes (Comité de Protection des Personnes Ile-de-France II, IRB registration number 00001073, protocol 20121300001072) and all participants gave written informed consent in line with the Declaration of Helsinki.

### Experimental Setup

The experimental setup used here to test our hypothesis was a modified version of the one employed in our previous studies [6], [7]. The system consisted of the following elements: a motion-analysis system with active markers (CODA; Charnwood Dynamics), that was used to measure the three-dimensional position of 27 infrared LEDs in real time (submillimeter accuracy, 200 Hz sampling frequency). Eight markers were distributed ∼10 cm apart on the surface of stereo virtual-reality goggles (nVisor sx60, NVIS) worn by the subjects, eight on the surface of tools (350 g, isotropic inertial moment around the roll axis) that were attached to each of the subjects’ hands and three attached to a fixed reference frame placed in the laboratory. For the goggles and the tools, a numerical model of the relative positions of the LEDs was implemented in advance, so that an optimal matching algorithm could be used to effectively and robustly estimate the position and the orientation of the object, even in case of partially hidden markers. We exploited the redundancy of the high number of markers on the helmet and on the tools to reduce errors in the position and orientation estimation, resulting in a standard error in the measured viewpoint orientation below the visual resolution of the goggles (0.078°). To minimize the effect of the noise and computational delays of the system, a predictive Kalman filter was applied to the angular coordinates of the objects.

The virtual environment consisted of a cylindrical horizontal tunnel whose walls were characterized by longitudinal marks parallel to the tunnel axis (Figure 1). These marks helped the subjects to perceive their own spatial orientation in the virtual world. Identification of the visual vertical was facilitated by the fact that the marks went from white on the ceiling to black on the floor. The real-time position and orientation of the goggles were then used to update, at 50 Hz, the visual scene viewed by the subject in the virtual environment. Data from markers on the tools attached to the hands were used when necessary (i.e. during training) to place a representation of the hand in the scene and to record the subjects’ movements.

### Experimental Procedure

In both INTRA and INTER experimental conditions the trial started with the subject’s head upright. Then automatic auditory commands asked the subject to raise one hand in front of him (the left or the right hand in the INTER or INTRA condition respectively). At this point the walls of the tunnel started changing color depending on the hand prono-supination angle. The color went from red to green as the hand approached the orientation that had to be memorized. Once the subject achieved the hand desired orientation, he or she had 2.5 sec to memorize it, after which the walls became insensitive to the hand orientation and the subject was instructed to lower the hand. After the target was acquired, subjects had 5 sec to tilt their head by 15°, to the right or to the left, depending on the trial. To guide subjects to the desired inclination of the head, audio feedback was provided: a sound with a left-right balance corresponding to the direction of the desired head inclination decreased in volume as the head approached 15°. If the subject was not able to turn off the sound within 5 sec, the trial was interrupted and was repeated later on. If he/she was able to reach the desired head inclination, after the 5 sec delay period that included the head roll movement, a signal was given to the subject to align the unseen right hand with the remembered target orientation and to validate the response by pressing a pedal with a foot.

*Sensory Conflict During Tilting of the Head:* Tracking the virtual-reality goggles was normally used to hold the visual scene stable with respect to the real world during movements of the head. But in half of the trials we generated a gradual, imperceptible conflict such that when the head tilted 15°, the subject received visual information corresponding to a rotation of 24°. The amplitude of the angle between the visual vertical and gravity varied proportionally (0.6 times) with the actual head tilt with respect to gravity, so that when the head was straight there was no conflict, and when the head was tilted to 15°, it was about 9°. At the end of the experiment the experimenter explicitly interviewed the subjects about the conflict perception. None of the subjects in this experiment reported to have noticed the tilt of the visual scene.

### Participants

After giving written informed consent, 16 subjects participated in this study: 8 male and 8 female, age 23±3 years (mean±standard deviation). All subjects performed both experimental conditions, and to compensate for possible order effects, half of them started with the INTRA condition and the other half with the INTER condition. For each of the two experimental conditions the subjects performed 56 trials: two for each combination of seven target orientations (−45°, −30°, −15°, 0°, +15°, +30°, +45°), two head inclinations (±15°), and two levels of conflict (no and yes).

### Data Analysis

We analyzed the recorded data in terms of errors (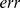) made at the moment of the response in aligning the hand with respect to the memorized target orientation. To quantify the specific effect of the sensory conflict in each condition, we first corrected for any global rotation of responses that might occur, for instance, due to possible Muller or Aubert effects (for review see [46]), independent from the tilt of the visual scene in the conflict situation. To do so, we subtracted from all values of 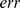 involving a head tilt to the right mean of such values obtained in the absence of sensory conflict and we did the same for leftward tilt, on a subject-by-subject basis. This allowed combining the trials with right and left head tilts. Next, for each value of 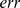 obtained with conflict we computed the relative deviation (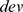) from the mean of all responses without conflict expressed as percentage of the expected deviation if only visual information was used, taking into account the actual amount of head tilt measured during the response phase of each trial.
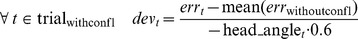
(1)


Finally, we computed the mean value of these relative deviations, which is a direct measure of the overall weight given to the visual versus other sources of information, for each subject and for each condition.
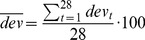
(2)


The variability of the performance of each individual subject in the trials without conflict was also evaluated. To robustly estimate each subject’s precision, despite the fact that only two responses could be used for each combination of target orientation and head inclination, the following procedure was used. First, the responses with the head tilted to the right and to the left were combined by compensating for possible Aubert-Muller effect, as reported above. Then, the variance, 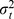, of the responses obtained for each of the 14 combinations of target and head orientation were combined as reported in the following equation to obtain the global standard deviation [47].
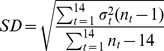
(3)


where 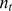 is the number of responses of the 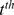 combination of target inclination and head orientation.

We used ANOVA for repeated measures on the values of response deviations and variability obtained with the methods described above to compare the INTRA and INTER conditions. The variability data, 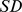, were transformed by the function 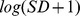, before performing the ANOVA [48]. To test whether the response deviations induced by the conflict were significantly different from the purely kinesthetic response (0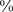), one-tailed Student’s t-test was used. To test whether the difference for response deviation between INTER and INTRA condition predicted by the models and experimentally observed differ significantly, two-tailed Student’s t-test was used.

### Mathematical Modeling

We evaluated our experimental results in the context of recent models by which sensory signals are combined and compared based on the principles of maximum likelihood. According to MLP, two signals (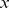 and 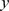) that are statistically independent will be optimally combined (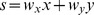) by assigning weights to each signal based on the relative variance between them:
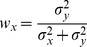
(4)

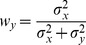
(5)


Similarly if three independent signals (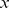, 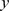 and 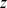) have to be combined (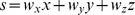) the optimal weights are given by the following equations:
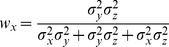
(6)

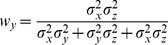
(7)

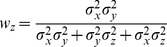
(8)


But if one signal is reconstructed from another, the two signals will not be independent. In this case the MLP equations used to determine the relative weights must be modified to take into account the covariance between signals, as follows. Let 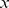 and 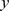 be two variables where each is the sum of a independent components 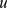 and 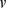, and a common component 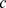.
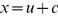
(9)

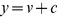
(10)


This additive formulation is representative of the computation required to shift two signals into a common reference frame. The variance of each variable is simply the sum of the variances of each component:
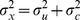
(11)

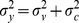
(12)


while the covariance between x and y is simply equal to the variance of the common component c:
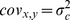
(13)


It can be easily demonstrated from basic principles (see Equations S1–S16 in online Supporting Information and [23]) that the relative weight, 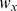 and 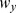, which minimizes the variance of the combination of 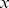 and 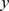: 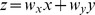 is:
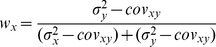
(14)

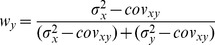
(15)


and substituting Eqs. 11, 12 and 13:
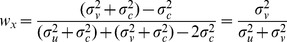
(16)

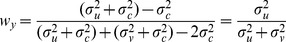
(17)


To optimally weight three quantities 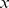, 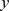 and 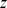, each of which is the sum of an independent component 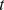, 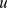 and 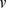, respectively, and a common component 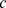, one finds a similar result:
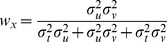
(18)

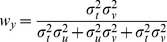
(19)

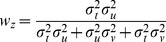
(20)


One can see from this derivation that computing the optimum weighting of 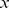 and 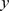, or of 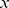, 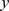 and 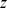, consists of computing the relative weights based on the variance of the independent components of each variable (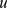, 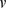, 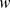), leaving out the variance of the common component 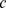. The standard deviation of the responses expected of each model computed is.
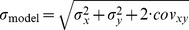
(21)or
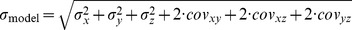
(22)depending on the structure of the model.

To illustrate the importance of taking into account co-variation of signals, and to compare with previous studies, we applied the MLP equations both ways, deliberately ignoring the co-variation between signals using Eqs. 4–5 for model A and Eqs. 6–8 for model B, and by correctly applying MLP in the case of co-variance using Eqs. 16–17 for model A’ and Eqs. 18–20 for model B’. In each case we attempted to fit the model parameters to the experimental data in order to estimate the ability of the different model formulations to predict the experimental results. Specifically, we searched for the set of free parameters, 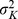, 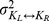, 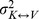 for each model (see Table 1), that, in conjunction with the modeling assumptions listed in the section entitled ‘theoretical modeling’ above, would minimize the difference between the actual and predicted responses deviations (Eq. 2 and Eq. 23 respectively), where the predicted deviation expressed as a percentage is given by:
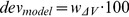
(23)and would minimize the difference between the actual and predicted response standard deviations (Eq. 3 and Eq. 21–22 respectively), simultaneously in both conditions (INTER and INTRA). The four data points were fit simultaneously by minimizing the weighted sum of the square of the differences between the experimental data and the model predictions for each data point, with the respective weights corresponding to the inverse of the squared confidence interval for each data point.

## Supporting Information

Supporting Information S1 **Equations for optimal sensory weighting in case of correlated signals.**
(PDF)
