# Supplementary material for: When Kinesthesia Becomes Visual: A Theoretical Justification for Executing Motor Tasks in Visual Space
Source: PLoS One. 2013 Jul 5;8(7):e68438. doi: 10.1371/journal.pone.0068438 (PMC3702599; doi:10.1371/journal.pone.0068438)
Supplement: Supporting Information S1 — Equations for optimal sensory weighting in case of correlated signals. (PDF) [file pone.0068438.s001.pdf]

Equations for optimal sensory weighting in case of correlated signals

Define z as the weighted sum of x and y:

$$z_i = w_x x_i + w_y y_i = \lambda x + (1-\lambda)y \tag{S1}$$

Without loss of generality, let z have a mean of zero. The variance of z is:

$$\sigma_z^2 = \frac{1}{n}\sum_{i=1}^{n} z_i^2 \tag{S2}$$

$$\sigma_z^2 = \frac{1}{n}\sum_{i=1}^{n}\left[\lambda x_i + (1-\lambda)y_i\right]^2 \tag{S3}$$

$$\sigma_z^2 = \frac{1}{n}\sum_{i=1}^{n}\left[\lambda^2 x_i^2 + (1-\lambda)^2 y_i^2 + 2\lambda(1-\lambda)x_i y_i\right] \tag{S4}$$

$$\sigma_z^2 = \frac{1}{n}\sum_{i=1}^{n}\left[\lambda^2 x_i^2 + (1-2\lambda+\lambda^2)y_i^2 + 2(\lambda-\lambda^2)x_i y_i\right] \tag{S5}$$

$$\sigma_z^2 = \lambda^2\frac{1}{n}\sum_{i=1}^{n} x_i^2 + (1-2\lambda+\lambda^2)\frac{1}{n}\sum_{i=1}^{n} y_i^2 + 2(\lambda-\lambda^2)\frac{1}{n}\sum_{i=1}^{n} x_i y_i \tag{S6}$$

$$\sigma_z^2 = \lambda^2\sigma_x^2 + (1-2\lambda+\lambda^2)\sigma_y^2 + 2(\lambda-\lambda^2)\operatorname{cov}(x,y) \tag{S7}$$

Find the λ that minimizes σ_z^2:

$$\frac{d\sigma_z^2}{d\lambda} = 2\lambda\sigma_x^2 + (2\lambda-2)\sigma_y^2 + 2(1-2\lambda)\operatorname{cov}(x,y) = 0 \tag{S8}$$

$$2\lambda(\sigma_x^2 + \sigma_y^2 - 2\operatorname{cov}(x,y)) - 2\sigma_y^2 + 2\operatorname{cov}(x,y) = 0 \tag{S9}$$

$$\lambda(\sigma_x^2 + \sigma_y^2 - 2\operatorname{cov}(x,y)) - \sigma_y^2 + \operatorname{cov}(x,y) = 0 \tag{S10}$$

$$\lambda(\sigma_x^2 + \sigma_y^2 - 2\operatorname{cov}(x,y)) = \sigma_y^2 - \operatorname{cov}(x,y) \tag{S11}$$

$$\lambda = \frac{\sigma_y^2 - \operatorname{cov}(x,y)}{\sigma_x^2 + \sigma_y^2 - 2\operatorname{cov}(x,y)} \tag{S12}$$

$$w_x = \lambda = \frac{\sigma_y^2 - \operatorname{cov}(x,y)}{(\sigma_x^2 - \operatorname{cov}(x,y) + (\sigma_y^2 - \operatorname{cov}(x,y)} \tag{S13}$$

$$w_y = (1-\lambda) = 1 - \frac{\sigma_y^2 - \operatorname{cov}(x,y)}{(\sigma_x^2 - \operatorname{cov}(x,y) + (\sigma_y^2 - \operatorname{cov}(x,y)} \tag{S14}$$

$$w_y = \frac{(\sigma_x^2 - \operatorname{cov}(x,y) + (\sigma_y^2 - \operatorname{cov}(x,y)}{(\sigma_x^2 - \operatorname{cov}(x,y) + (\sigma_y^2 - \operatorname{cov}(x,y)} - \frac{\sigma_y^2 - \operatorname{cov}(x,y)}{(\sigma_x^2 - \operatorname{cov}(x,y) + (\sigma_y^2 - \operatorname{cov}(x,y)} \tag{S15}$$

$$w_y = \frac{\sigma_x^2 - \operatorname{cov}(x,y)}{(\sigma_x^2 - \operatorname{cov}(x,y) + (\sigma_y^2 - \operatorname{cov}(x,y)} \tag{S16}$$
